# Microlithiasis of Seminal Vesicles and Severe Oligoasthenospermia in Pulmonary Alveolar Microlithiasis (PAM): Report of An Unusual Sporadic Case

**DOI:** 10.22074/ijfs.2015.4218

**Published:** 2015-04-21

**Authors:** Giuseppe Castellana, Domenico Carone, Marco Castellana

**Affiliations:** 1 District Health Center, ASL BA, via de Amicis, Conversano (70014), Bari, Italy; 2 Center of Human Reproduction and Andrology (CREA), Via Scoglio del Tonno 79/81, Taranto (74120), Italy; 3 University of Bari, Piazza Giulio Cesare 11, Bari (70124), Italy

**Keywords:** Seminal Vesicles, Microlithiasis, Oligoasthenospermia, Infertility, Pulmonary Alveolar Microlithiasis

## Abstract

Pulmonary alveolar microlithiasis (PAM) is classified as an elective dysmetabolic thesaurotic pneumoalveolitis and characterized by the presence within the alveoli of the lungs of myriad of tiny calculi. The classic presentation of the chest radiography is unmistakable with multiple small "sand-like" opacities diffusely involving both lung fields.

We present a case of male infertility for hypoposia and severe oligoasthenospermia in a young patient with recurrent haematuria and small calcifications in the seminal vesicles similar to pulmonary microliths. PAM was diagnosed on routine chest radiography, com- puter tomography (CT), transbronchial biopsy and bronchoalveolar lavage (BAL).

## Introduction

Pulmonary alveolar microlithiasis ( PAM ) ( OMIM 265100 ) is a rare disease caused by the mutation of the *SLC34A2* gene, encoding the type IIb sodiumphosphate co-transporter in alveolar type II cells. It is characterized by the diffuse presence in the pulmonary alveoli of microliths ( calcified lamellar deposits measuring 0.01-3 mm made of calcium phosphate ) and has a typical radiographic appearance featuring diffuse "sand-like" micronodules (1,2). The extension and severity of the disease can best be assessed by high resolution computerized tomography scan ( HRCT ). A radiological classification of the disease, subdivided into four phases of evolution, has been proposed (3). 

PAM is present worldwide and at the most recent review of the literature, a total of 576 cases had been reported. As regards distribution in the continents, it has most frequently been described in Europe, followed by Asia, especially Asia Minor. The nation with the highest number of reported cases is Turkey, followed by Italy and then the United States of America, India, Russia and Germany (4). 

Cases of PAM are defined as "familial" when two or more members of a family ( usually two or three, but exceptionally four, five or even six ) are found to be affected by the disease, and "sporadic" when family history is negative. Sporadic cases are prevalent in the male sex, whereas familial cases are much more frequently observed in females. The transmission of the disease is autosomal recessive (5). 

PAM is not easily described as regards its clinical course, including the initial phase, evolution and stabilization. The onset age varies from the neonatal period to the geriatric. On the admission, more than half the patients are asymptomatic, while others complain dyspnoea, cough, chest pain, fever or sputum, cyanosis and finger clubbing are also reported. The illness may remain static as regards both symptoms and radiographic findings, or it may worsen over time, leading to pulmonary fibrosis, respiratory failure and chronic pulmonary heart disease (4,6). 

In most of the cases of PAM described in the literature, the disease is confined to the lungs and similar lesions have almost never been described in other organs and tissues. The only exceptions are the following ones: pleura, sympathetic ganglia, epididymis, seminal vesicles and prostate (7,12). 

Calcifications have also been reported in other organs, such as urinary and biliary tract, but they do not seem to have the typical aspects of microliths (11,13,16). 

Separate considerations should be made for testicular microlithiasis ( TM ), which is not rare, it has a prevalence of 0.6-9% in the population and is associated with 1% of idiopathic infertility cases. Analyzing 15 subjects with diffuse bilateral TM, Corut identified 2 patients with 2 rare variants in the heterozygous state for *SLC34A2*: the first variant was a synonymous, the second was noncoding. He also evaluated seven male patients with PAM, but none had positive findings when investigated for TM (1). These results seem to suggest a different aetiology for PAM and TM. 

We report here a patient affected by microlithiasis of the seminal vesicles and severe oligoasthenospermia associated with PAM. 

## Case Report

**Fig.1:**
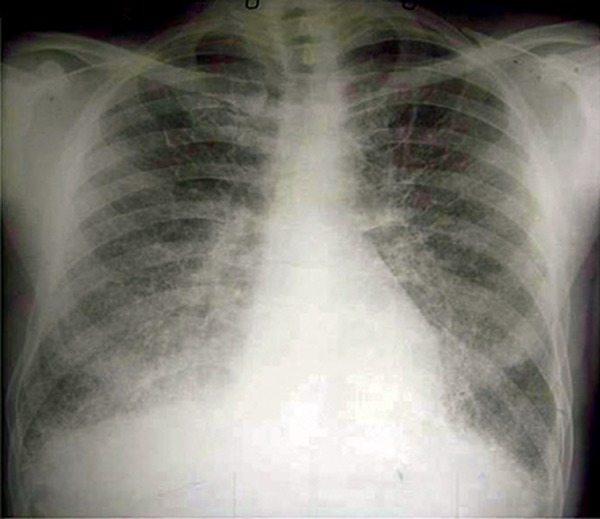
Chest X-ray: parenchyma puntiform shadows bilaterally diffuse in lungs, predominantly in the middle and lower fields.

**Fig.2:**
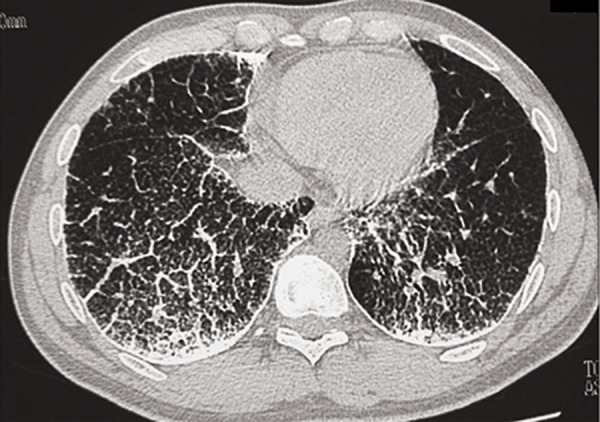
Chest HRCT: bilateral calcific micronodules diffusely involving the parenchyma and diffuse interstitial pattern ( septal thickening ). HRCT; High resolution computerized tomography scan.

A 32-year-old male underwent medical investigations for recurrent haematuria. Chest X-rays showed diffuse interstitial disease and the patient was referred for a pneumology examination. He denied ever suffering from respiratory troubles and was a non smoker. Routine blood tests were normal, as were spirometry and blood-gas analysis. Close examination of the chest X-rays (Fig .1) revealed a diffuse, fine, sand-like deposit involving both entire lungs, but especially the bases. HRCT (Fig .2), broncho-alveolar lavage ( BAL ) and transbronchial biopsy were performed that demonstrated the characteristic microliths. A genetic study on the *SLC34A2* gene was not carried. Screening by chest X-rays of the parents and two brothers yielded negative results. 

**Fig.3:**
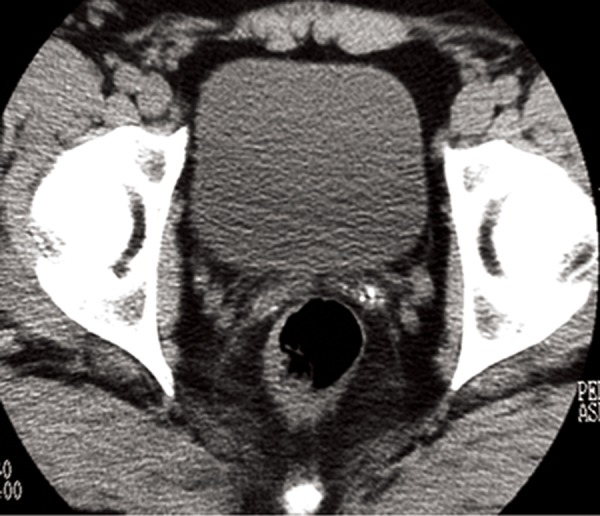
Pelvic HRCT: microliths in the seminal vesicles. HRCT; High resolution computerized tomography scan.

**Fig.4:**
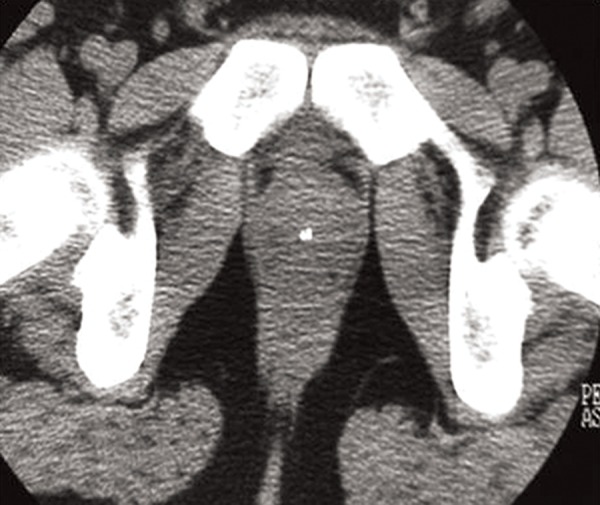
Pelvic HRCT: calcification in the prostate. HRCT; High resolution computerized tomography scan.

Transrectal ultrasound showed minute calcifications of the seminal vesicles similar to those in the lungs and a calcification in the prostate. The patient was married and after two years of infertility despite complete unprotected sexual relations, he underwent a spermiogram that revealed evidence of hypofertility with a low volume of ejaculate ( < 1 ml ) and a sperm count ranging from 3×10 ^6^ to 15×10^6^/ml, with low levels of fructose in the seminal plasma. Oligoasthenospermia and hypoposia due to distal obstruction of the seminal tract were diagnosed. Pelvic CT scans (Figs.3, 4) at the level of the seminal vesicles confirmed the ultrasonography (US) findings, demonstrating small radiopaque areas resembling the pulmonary microliths and a calcification in the prostate measuring 4 mm with a different appearance from the microliths. The patient gave an informed consent for the case report.

## Discussion

In this paper, we report the diagnosis of microlithiasis in seminal vesicles and PAM in a patient with recurrent haematuria, infertility and no respiratory symptoms. 

For many years, the aetiology of PAM was a matter of debate, until the discovery of *SLC34A2* gene in 2006 (1). This finding raises a new question: could PAM really be a systemic disease and its localization to the male genital tract cause infertility? In 1967, O’Neill et al. reported a family pedigree showing relative infertility in one male member affected by PAM: he had only 2 children, in contrast to numerous progeny of the other siblings. Since he died at the age of 37 and no contraceptive practices were used, a large number of offspring would be expected in that setting (17). Sandhyamani et al. (18) reported a 27-year-old man with PAM and infertility caused by focal bilateral testicular atrophy related to seminal vesicle microliths. Chatterji et al. (19) reported a 40-year-old male with PAM and primary infertility caused by azoospermia. Kanat et al. (9) reported a 37-year-old man with PAM and epididymis and periurethral microliths causing obstructive azoospermia. 

In the present case, small calcifications measuring approximately 1 mm, similar in appearance to those in the lungs, were diffusely distributed in the seminal vesicles. A remarkably variable sperm count with low level of fructose is considered as typical findings in forms of oligoasthenospermia due to distal obstruction of the seminal tract; therefore, it is assumed that there could be a correlation between the microlithiasis of the seminal vesicle and seminal data. 

This clinical case is unusual inasmuch as it is only the third case of association of microlithiasis of the lungs and seminal vesicles. Therefore, it has prompted us to raise doubts about the exclusive localization of microlithiasis in the pulmonary alveoli, as has always been believed, and to support the hypothesis that the disease could be systemic or at any rate could also involve the male genital apparatus leading to infertility. This peculiar association needs to be further studied and suggests the need to make a particularly close examination of the chest X-ray in some cases of male infertility. 

If PAM is suspected, the best diagnostic schedule is the association of BAL and HRCT, as the first investigation can document the diagnosis, while the second provides further information about the degree of inflammation and/or fibrosis or calcification of the interstices. Nevertheless, standard radiography of the chest is quite reliable enough when there is already a known case in the family. 

Identification of the *SLC34A2* gene mutation clinches the diagnosis. When the diagnosis is established, the examination of the family of the index patient is mandatory (2). 
